# Extreme Levels of HbA1c Increase Incident ESRD Risk in Chinese Patients with Type 2 Diabetes: Competing Risk Analysis in National Cohort of Taiwan Diabetes Study

**DOI:** 10.1371/journal.pone.0130828

**Published:** 2015-06-22

**Authors:** Li-Na Liao, Chia-Ing Li, Chiu-Shong Liu, Chiu-Ching Huang, Wen-Yuan Lin, Jen-Huai Chiang, Cheng-Chieh Lin, Tsai-Chung Li

**Affiliations:** 1 Department of Public Health, College of Public Health, China Medical University, Taichung, Taiwan; 2 Department of Medical Research, China Medical University Hospital, Taichung, Taiwan; 3 School of Medicine, College of Medicine, China Medical University, Taichung, Taiwan; 4 Department of Family Medicine, China Medical University Hospital, Taichung, Taiwan; 5 Kidney Institute and Division of Nephrology, Department of Internal Medicine, China Medical University Hospital, Taichung, Taiwan; 6 Management Office for Health Data, China Medical University Hospital, Taichung, Taiwan; 7 Research Center for Chinese Medicine & Acupuncture, China Medical University, Taichung, Taiwan; 8 Graduate Institute of Biostatistics, College of Public Health, China Medical University, Taichung, Taiwan; 9 Department of Healthcare Administration, College of Health Science, Asia University, Taichung, Taiwan; Heinrich-Heine University, Faculty of Medicine, GERMANY

## Abstract

**Background:**

Whether HbA1c is a predictor of end-stage renal disease (ESRD) in type 2 diabetes patients remains unclear. This study evaluated relationship between HbA1c and ESRD in Chinese patients with type 2 diabetes.

**Methods:**

Patients aged ≥ 30 years who were free of ESRD (n = 51 681) were included from National Diabetes Care Management Program from 2002–2003. Extended Cox proportional hazard model with competing risk of death served to evaluate association between HbA1c level and ESRD.

**Results:**

A total of 2613 (5.06%) people developed ESRD during a follow-up period of 8.1 years. Overall incidence rate of ESRD was 6.26 per 1000 person-years. Patients with high levels of HbA1c had a high incidence rate of ESRD, from 4.29 for HbA1c of  6.0%–6.9% to 10.33 for HbA1c ≥ 10.0% per 1000 person-years. Patients with HbA1c < 6.0% particularly had a slightly higher ESRD incidence (4.34 per 1000 person-years) than those with HbA1c  of 6.0%–6.9%. A J-shaped relationship between HbA1c level and ESRD risk was observed. After adjustment, patients with HbA1c < 6.0% and ≥ 10.0% exhibited an increased risk of ESRD (HR: 1.99, 95% CI: 1.62–2.44; HR: 4.42, 95% CI: 3.80–5.14, respectively) compared with those with HbA1c of 6.0%–6.9%.

**Conclusions:**

Diabetes care has focused on preventing hyperglycemia, but not hypoglycemia. Our study revealed that HbA1c level ≥ 7.0% was linked with increased ESRD risk in type 2 diabetes patients, and that HbA1c < 6.0% also had the potential to increase ESRD risk. Our study provides epidemiological evidence that appropriate glycemic control is essential for diabetes care to meet HbA1c targets and improve outcomes without increasing the risk to this population. Clinicians need to pay attention to HbA1c results on diabetic nephropathy.

## Introduction

Diabetes has become one of the most common causes of end-stage renal disease (ESRD) in numerous countries, and a 44.6%, 44.5%, and 43.7% incidence of ESRD in patients is caused by diabetes in Japan, Taiwan, and the United States, respectively [1]. Because of the alarming rise in the number of diabetes cases worldwide [2], the ESRD population is increasing. The prevalence and incidence of ESRD is increasing rapidly in Taiwan [3]. The total number of regular dialysis patients increased by 26.5% from 52 081 in 2006, to 65 883 in 2010 [4, 5]. The rising number of ESRD patients requiring dialysis therapy or transplantation is a population health problem, which places a substantial burden on medical and health resources [1].

Hyperglycemia is the most crucial factor in the progression of microvascular complications of diabetes, including nephropathy. The American Diabetes Association (ADA) recommended that target HbA1c should be below or around 7.0% [6]. A longitudinal study demonstrated that controlling HbA1c < 7.0% reduced new-onset microalbuminuria risk by 27.1% in a cohort of type 2 diabetes patients with normoalbuminuria [7]. Several studies have focused on the association between glycemic control and early onset diabetic nephropathy (DN), defined by micro- or macroalbuminuria for clinical renal outcomes in type 2 diabetes patients [8–11]. Several studies have reported that strict glycemic control intervention reduced the risk of microalbuminuria and macroalbuminuria [8, 9], whereas others have not [10, 11].

Previous studies have evaluated the associations between intervention targeting strict glycemic control and substantially clinical renal outcomes, such as ESRD requiring dialysis therapy in type 2 diabetes patients [8–11]. However, the findings on the relationships between strict glycemic control intervention and ESRD in these studies are conflicting. Perkovic et al observed that intensive glucose control intervention significantly reduced ESRD risk [9], but no significant effect of intensive glycaemia therapy on ESRD has been observed in other studies [8, 10, 11]. The primary aim of a randomized clinical trial (RCT) is to assess the intervention effect of strict glycemic control on ESRD outcome, not the association between HbA1c level and ESRD incidence. Among Canadian patients with diabetes and chronic kidney disease (CKD), a U-shaped relationship between HbA1c levels and all-cause mortality was observed, but not in ESRD patients [12]. A South Korea study revealed that ESRD risk in HbA1c of 6.50%–7.49% and≥7.50% were significantly increased compared with HbA1c of < 6.50%; because of the limited sample size and a hospital-based study design, the authors could not evaluate whether a lower level of HbA1c increased or decreased ESRD risk [13].

Whether extreme levels of HbA1c increase the risk of ESRD incidence in the Han Chinese population has not been reported. The effect of HbA1c on ESRD among type 2 diabetes patients warrants further investigation. Therefore, we estimated the incidence of ESRD according to HbA1c levels, and evaluated whether a J- or U-shaped relationship between HbA1c levels and ESRD risk exists in a large cohort of ethnic Chinese patients with type 2 diabetes enrolled in the National Diabetes Care Management Program (NDCMP).

## Materials and Methods

### Study Participants

Our study population from a national cohort of Taiwan Diabetes Study comprised 62 656 diabetic patients enrolled in the NDCMP from 2002–2003 in Taiwan. The date of entry into the NDCMP was defined as the index date. The NDCMP provided a set of integrated examinations and performance measures for patients. At the beginning of entering the NDCMP, patients underwent a series of medical tests for blood, urine, and body measurements; and patients were required to complete a standardized and computerized questionnaire administered by a case management nurse to record the previous status of their medication and lifestyle behaviors. This nationally managed care for diabetes patients was established by the Bureau of National Health Insurance (BNHI) in November 2001. Its goal is to integrate related departments, expertise, and hospitals at diverse levels to provide continual, approachable, and high-quality care for diabetes patients. All patients diagnosed with diabetes (International Classification of Diseases, Ninth Revision, Clinical Modification (ICD-9-CM); code 250) based on ADA criteria were invited to participate in the NDCMP. In this study, patients with type 1 (ICD-9-CM codes: 250.x1/x3) or gestational (ICD-9-CM codes: 648.83) diabetes (n  =  2072), aged less than 30 years (n  =  651), and those missing diabetes-related variables information (n  =  7091) were excluded. We also excluded patients with a diagnosis of ESRD at baseline or incidence ESRD cases within 1 year of the index date to eliminate cause-and-effect (n  =  317), along with patients who died or withdrew from the National Health Insurance (NHI) program within the first year after participating in the NDCMP (n  =  844). Finally, 51 681 participants were included for further analysis. This study was approved by the Ethical Review Board of China Medical University Hospital. Patient records and information were anonymized and de-identified prior to analysis.

### Baseline Assessments

The key independent variable was baseline HbA1c. According to the clinical criteria of HbA1c, the participants were grouped into 6 categories: < 6.0%, 6.0%–6.9%, 7.0%–7.9%, 8.0%–8.9%, 9.0%–9.9%, and≥10.0%. Covariates at baseline consisted of sociodemographic factors, lifestyle behaviors, biochemical variables, diabetes-related variables, and comorbidities including age, sex, smoking status (no, yes), alcohol consumption (yes, no), fasting plasma glucose (FPG), total cholesterol, triglycerides, high-density lipoprotein cholesterol (HDL-C), low-density lipoprotein cholesterol (LDL-C), serum creatinine, duration of diabetes, type of antidiabetes medication (diet and exercise or oral monotherapies, oral polypharmacotherapies, and insulin treatments), antihypertensive medication (yes, no), obesity (body mass index≥27 kg/m^2^), diabetic ketoacidosis (DKA) (yes, no), hyperosmolar non-ketoacidosis (HHNK) (yes, no), severe hypoglycemia (yes, no), and neuropathy (yes, no). Regarding DKA, HHNK, severe hypoglycemia, and neuropathy, we used data collected in the NDCMP, which used the standardized and computerized questionnaire to record whether patients had ever occurred each disease event. Renal function was evaluated using glomerular filtration rate (GFR); estimated GFR (eGFR) was calculated using the Chronic Kidney Disease Epidemiology Collaboration equation: eGFR (mL/min/1.73 m^2^)  =  141×min (Scr/κ, 1)^α^ × max (Scr/κ, 1)^−1.209^ × 0.993^Age^×1.018 (if female) ×1.159 (if black), where Scr is serum creatinine, κ is 0.7 for women and 0.9 for men, α is -0.329 for women and -0.411 for men, min indicates the minimum of Scr/κ or 1, and max indicates the maximum of Scr/κ or 1 [14]. After a 12-hr overnight fast, blood was drawn from an antecubital vein in the morning and sent for analysis within 4-hr post-collection.

Baseline comorbidities included hypertension (ICD-9-CM codes: 401–405), hyperlipidemia (ICD-9-CM code: 272), cerebral vascular accident (CVA; ICD-9-CM codes: 430–438), coronary artery disease (CAD; ICD-9-CM codes: 410–413, 414.01–414.05, 414.8, and 414.9), congestive heart failure (CHF; ICD-9-CM codes: 428, 398.91, 402.01, 402.11, and 402.91), and cancer (ICD-9-CM codes: 140–165, 170–175, 179–200, 202, 203, 210–213, 215–229, 235–239, 654.1, and 654.10–654.14). A diabetic patient was defined as having one of these comorbidities by using at least one inpatient or three ambulatory claims data which were collected for 1-year period prior to cohort entry. Data were from the National Health Insurance Research Database (NHIRD), which is affiliated with the Taiwan NHI program initiated on March 1, 1995 and maintained by the National Health Research Institutes. In 2007, more than 98% of Taiwan’s population of 23 million were covered by the NHI program [15]. To protect privacy, data on patient identities were scrambled cryptographically by the NHIRD. The validity of claims data is ensured by the BNHI; claims data are examined including procedures examination, expert review, on-site examination, and files analysis [16]. In the expert review stage, a random sample for every 50–100 ambulatory and inpatient claims in each hospital and clinic is examined by experts with clinical experiences, based on medical theory, medical requirement, treatment priorities, etc. A severe penalty is enforced for false diagnosis reports, and the misclassification of baseline comorbidities is rare.

### Primary Outcome Ascertainment

Our outcome of interest was ESRD defined by requiring dialysis at least 3 months. These incident ESRD cases (ICD-9-CM code: 585) were identified from the NHIRD Registry for Catastrophic Illness Patient Database excluding patients with acute kidney failure. The physician diagnosed ESRD by performing a physical examination and tests to check renal function. Tests may include blood tests, urine tests, renal imaging, and kidney biopsy. Patients with ESRD approved for catastrophic illness registration cards are those who have received hemodialysis or peritoneal dialysis for over 3 months. Insured ESRD patients with catastrophic illness registration cards are not required to pay medical expenses when they seek health care for ESRD-related conditions.

### Statistical Analysis

The baseline period was 2002–2003. Participants were followed until the development of ESRD, death, or the end of 2011. The baseline demographic and clinical characteristics of the participants in the NDCMP were examined according to HbA1c levels. Continuous variables were reported as the mean±standard deviation (SD), and categorical variables were tallied as a number and percentage. Bivariate statistical methods (eg, one-way analysis of variance, Chi-square test) were used to explore data features. Incident ESRD density rates per HbA1c level were estimated, and Kaplan-Meier cumulative incidence curves were derived. The extended Cox proportional hazards model using the Lunn-McNeil approach was applied as a modified Cox proportional hazards model that considered competing risks and allowed for multivariable adjustment [17]. The extended Cox proportional hazard models with the competing risk of death were used to evaluate the association between HbA1c level and incident ESRD. Hazard ratios (HRs) and their 95% confidence intervals (CIs) were calculated. Three multivariate models were built: first, we adjusted for age and sex; second, we additionally adjusted for diabetes duration, smoking, drinking, obesity, hypertension, antihypertensive medication, hyperlipidemia, types of antidiabetes medication, and baseline eGFR; and, third, we additionally adjusted for neuropathy, severe hypoglycemia, DKA, HHNK, CVA, CAD, CHF, and cancer. Two forms of sensitivity analysis were performed. One was conducted to investigate the potential bias caused by the existence of comorbidities by excluding those patients with DKA, HHNK, severe hypoglycemia, CVA, CAD, and CHF. The other was conducted to increase the precision of HbA1c measurements by using the average value of all HbA1c measurements within 1 year since the index date. The analyses were conducted using SAS version 9.3 (SAS Institute Inc., Cary, NC), and significance was set at a 2-sided *P* < .05.

## Results

The demographic and clinical characteristics of the 51 681 NDCMP participants are summarized according to baseline HbA1c levels in Table 1. The mean age of the participants was 61 years. In our study, 2613 (5.06%) patients were diagnosed with ESRD during a mean follow-up period of 8.1 years (median: 8.8 y, IQR: 8.2–9.2 y). The overall incidence density rate (IDR) of ESRD was 6.26 per 1000 person-years. Furthermore, the overall IDR of death was 24.68 per 1000 person-years, and patients with baseline HbA1c level≥10.0% had the highest mortality, 27.33 per 1000 person-years. The mean ages of the 6 HbA1c groups ranged from 58 to 63 years. Patients with baseline HbA1c≥10.0% had a high smoking prevalence and high mean values of FPG, total cholesterol, triglycerides, HDL-C, and LDL-C.

**Table 1:** Characteristics of study subjects stratified by HbA1c levels.

Characteristic^a^		HbA1c					
	Total (n = 51681)	<6.0% (n = 4437; 8.59%)	6.0–6.9% (n = 10402; 20.13%)	7.0–7.9% (n = 11134; 21.54%)	8.0–8.9% (n = 8856; 17.14%)	9.0–9.9% (n = 6517; 12.61%)	≥10.0% (n = 10335; 20.00%)
ESRD	2613 (5.06)	155 (3.49)	364 (3.50)	465 (4.18)	422 (4.77)	365 (5.60)	842 (8.15)
**Sociodemogrpahic factors**							
Men	24367 (47.15)	2435 (54.88)	5128 (49.30)	5178 (46.51)	3927 (44.34)	2818 (43.24)	4881 (47.23)
Age (years)	60.72 ± 11.28	62.6 ± 11.92	62.4 ± 11.23	61.64 ± 11.04	60.74 ± 10.99	59.57 ± 11.02	57.91 ± 11.08
Age ≥65 years	19800 (38.31)	2110 (47.55)	4653 (44.73)	4613 (41.43)	3334 (37.65)	2205 (33.83)	2885 (27.91)
**Lifestyle behaviors**							
Smoking	8080 (15.63)	637 (14.36)	1484 (14.27)	1610 (14.46)	1337 (15.10)	1055 (16.19)	1957 (18.94)
Alcohol drinking	4438 (8.59)	428 (9.65)	851 (8.18)	955 (8.58)	746 (8.42)	536 (8.22)	922 (8.92)
**Biochemicla variables**							
FPG (mg/dL)	174.89 ± 67.32	125.74 ± 40.22	138.62 ± 44.63	155.08 ± 44.78	176.99 ± 52.35	198.67 ± 60.45	237.04 ± 78.40
Total cholesterol (mg/dL)	195.52 ± 39.86	184.92 ± 37.09	189.81 ± 36.81	193.30 ± 37.93	196.54 ± 39.30	198.86 ± 40.48	205.24 ± 43.56
Triglycerides (mg/dL)^b^	142.59 ± 1.82	122.73 ± 1.75	130.32 ± 1.77	137.00 ± 1.77	146.94 ± 1.80	151.41 ± 1.84	157.59 ± 1.92
HDL-C (mg/dL)	45.90 ± 13.19	45.66 ± 13.24	45.99 ± 13.14	45.71 ± 13.00	45.74 ± 12.86	45.49 ± 12.87	46.51 ± 13.84
LDL-C (mg/dL)	118.09 ± 31.13	111.40 ± 29.48	115.03 ± 30.08	117.12 ± 30.26	119.11 ± 31.11	119.46 ± 31.54	123.34 ± 32.57
Creatinine (mg/dL)	1.03 ± 0.52	1.10 ± 0.52	1.07 ± 0.54	1.05 ± 0.51	1.02 ± 0.51	1.00 ± 0.46	0.98 ± 0.52
eGFR (ml/min/1.73m²)	74.62 ± 22.24	70.57 ± 21.71	71.95 ± 21.50	72.96 ± 21.92	74.79 ± 22.08	76.33 ± 22.41	79.60 ± 22.57
eGFR <60 ml/min/1.73m²	13438 (26.00)	1368 (30.83)	2994 (28.78)	3128 (28.09)	2261 (25.53)	1587 (24.35)	2100 (20.32)
CKD stages							
Stage 1	2485 (4.81)	188 (4.24)	480 (4.61)	515 (4.63)	435 (4.91)	325 (4.99)	542 (5.24)
Stage 2	4128 (7.99)	430 (9.69)	1016 (9.77)	978 (8.78)	728 (8.22)	412 (6.32)	564 (5.46)
Stage 3a	8395 (16.24)	840 (18.93)	1838 (17.67)	1939 (17.42)	1409 (15.91)	1016 (15.59)	1353 (13.09)
Stage 3b	3592 (6.95)	367 (8.27)	816 (7.84)	856 (7.69)	617 (6.97)	414 (6.35)	522 (5.05)
Stage 4	1201 (2.32)	131 (2.95)	273 (2.62)	268 (2.41)	204 (2.30)	136 (2.09)	189 (1.83)
Stage 5	250 (0.48)	30 (0.68)	67 (0.64)	65 (0.58)	31 (0.35)	21 (0.32)	36 (0.35)
**Diabetes-related variables**							
Diabetes durations (years)	6.49 ± 6.56	4.73 ± 5.77	5.71 ± 6.11	6.87 ± 6.74	7.22 ± 6.78	7.37 ± 6.83	6.46 ± 6.54
Type of antidiabetes medication							
Diet/exercise or oral monotherapies	10989 (21.26)	1973 (44.47)	3551 (34.14)	2350 (21.11)	1274 (14.39)	772 (11.85)	1069 (10.34)
Oral polypharmacotherapies	33008 (63.87)	2217 (49.97)	6114 (58.78)	7529 (67.62)	6177 (69.75)	4355 (66.83)	6616 (64.02)
Insulin treatments	7684 (14.87)	247 (5.57)	737 (7.09)	1255 (11.27)	1405 (15.86)	1390 (21.33)	2650 (25.64)
**Drug-related variables**							
Antihypertensive medication	19219 (37.19)	1725 (38.88)	4225 (40.62)	4451 (39.98)	3416 (38.57)	2355 (36.14)	3047 (29.48)
**Diabetes-related diseases**							
Obesity (BMI ≥27 kg/m^2^)	18538 (35.87)	1556 (35.07)	3839 (36.91)	4201 (37.73)	3290 (37.15)	2422 (37.16)	3230 (31.25)
BMI (kg/m^2^)	25.59 ± 3.79	25.57 ± 3.84	25.71 ± 3.71	25.78 ± 3.69	25.78 ± 3.80	25.65 ± 3.82	25.06 ± 3.90
Hypertension	22470 (43.48)	2055 (46.32)	4867 (46.79)	5261 (47.25)	4016 (45.35)	2773 (42.55)	3498 (33.85)
SBP (mmHg)	134.59 ± 17.92	134.89 ± 17.87	134.55 ± 17.47	135.3 ± 17.60	135.01 ± 17.67	135.19 ± 18.35	133.01 ± 18.55
DBP (mmHg)	80.05 ± 10.73	79.38 ± 11.07	79.61 ± 10.53	80.03 ± 10.51	80.14 ± 10.59	80.71 ± 10.81	80.31 ± 11.04
Hyperlipidemia	12354 (23.90)	953 (21.48)	2649 (25.47)	2884 (25.90)	2198 (24.82)	1598 (24.52)	2072 (20.05)
Neuropathy	5122 (9.91)	359 (8.09)	880 (8.46)	973 (8.74)	949 (10.72)	764 (11.72)	1197 (11.58)
Severe hypoglycemia	2132 (4.13)	242 (5.45)	467 (4.49)	475 (4.27)	366 (4.13)	260 (3.99)	322 (3.12)
DKA	719 (1.39)	65 (1.46)	126 (1.21)	123 (1.10)	122 (1.38)	98 (1.50)	185 (1.79)
HHNK	1103 (2.13)	99 (2.23)	189 (1.82)	209 (1.88)	198 (2.24)	143 (2.19)	265 (2.56)
CVA	2732 (5.29)	292 (6.58)	557 (5.35)	572 (5.14)	489 (5.52)	372 (5.71)	450 (4.35)
Coronary artery disease	4520 (8.75)	383 (8.63)	940 (9.04)	1053 (9.46)	842 (9.51)	588 (9.02)	714 (6.91)
Congestive heart failure	1247 (2.41)	112 (2.52)	271 (2.61)	296 (2.66)	198 (2.24)	171 (2.62)	199 (1.93)
Cancer	1072 (2.07)	97 (2.19)	231 (2.22)	229 (2.06)	209 (2.36)	128 (1.96)	178 (1.72)
Death^c^	10445 (20.21)	939 (21.16)	2000 (19.23)	2144 (19.26)	1750 (19.76)	1334 (20.47)	2278 (22.04)
IDR of death (per 1,000 person-years)^d^	24.68	25.98	23.33	23.34	24.03	25.06	27.33

Data were presented as mean±SD for continuous variables or n (%) for categorical variables. IDR: incidence density rate; FPG: fasting plasma glucose; HDL-C: high-density lipoprotein cholesterol; LDL-C: low-density lipoprotein cholesterol; eGFR: estimated glomerular filtration rate; BMI: body mass index; SBP: systolic blood pressure; DBP: diastolic blood pressure; DKA: diabetic ketoacidosis; HHNK: hyperglycemic hyperosmolar nonketotic coma; CVA: cerebral vascular accident.

a: All *P*-values were less than .05 for comparing HbA1c groups.

b: Geometric mean was presented.

c: The number of death included patients who died either with or without ESRD during the follow-up period.

d: IDR = number of incidence cases/person-years*1000.

**Fig 1:**
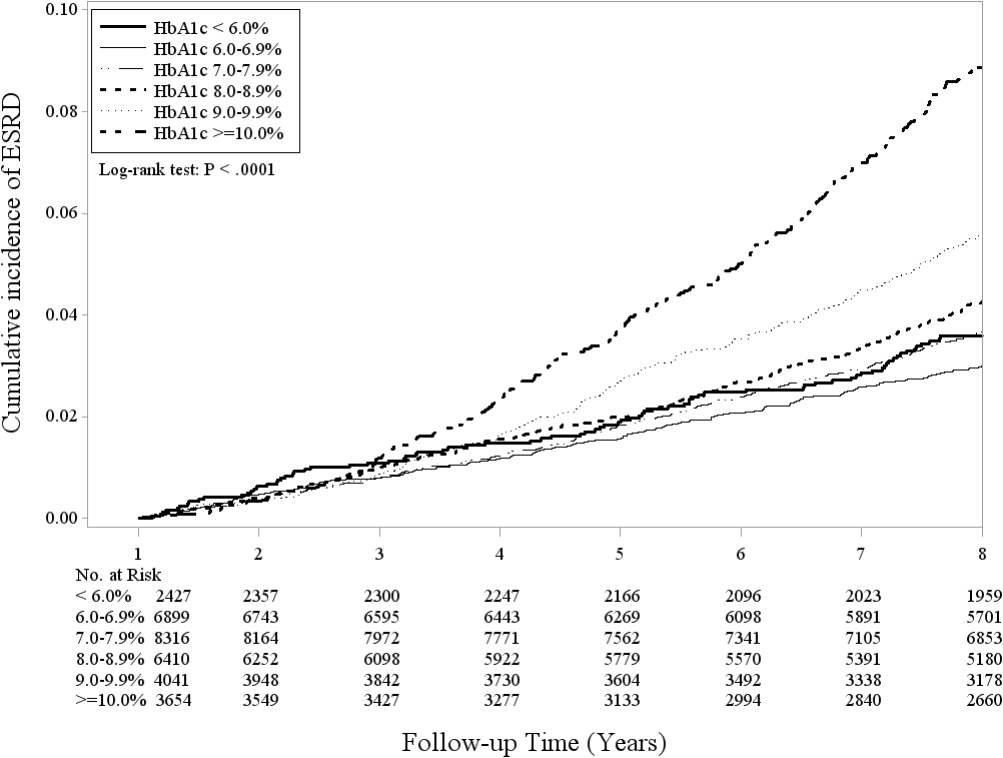
Cumulative incidence curves of ESRD according to clinical criteria of HbA1c levels.

Table 2 shows the IDRs of ESRD according to HbA1c levels, HRs of ESRD, and their 95% CIs. Patients with high levels of baseline HbA1c had high IDRs of ESRD, from 4.34 for HbA1c < 6.0% to 10.33 for HbA1c≥10.0% per 1000 person-years. A J-shaped relationship between HbA1c and incident ESRD risk was observed. Patients with HbA1c < 6.0% and≥10.0% were at a 1.99- and 4.42-fold greater risk of ESRD (95% CI: 1.62–2.44, *P* < .001 and 95% CI: 3.80–5.14, *P* < .001, respectively) than were patients with HbA1c levels of 6.0%–6.9% after considering age, sex, diabetes duration, lifestyle behaviors, eGFR, medications, and comorbidities. In a sensitivity analysis, we observed similar results after excluding patients with DKA, HHNK, severe hypoglycemia, CVA, CAD, and CHF; the adjusted HRs and their 95% CIs were 2.26 (1.76–2.92), 1.94 (1.59–2.37), 2.33 (1.90–2.85), 2.98 (2.42–3.66), and 4.99 (4.15–6.01) in HbA1c < 6.0%, 7.0%–7.9%, 8.0%–8.9%, 9.0%–9.9%, and≥10.0%, respectively, compared with that in HbA1c 6.0%–6.9%. Furthermore, to reduce the random error of one HbA1c measurement, we used the mean value of HbA1c measurements obtained within 1 year as the predictor of ESRD risk (n  =  31 747). We also observed a J-shaped relationship between HbA1c and ESRD risk; that is, the adjusted HRs and their 95% CIs were 2.20 (1.70–2.85), 1.80 (1.47–2.19), 2.10 (1.72–2.56), 2.66 (2.16–3.29), and 4.32 (3.53–5.28) in HbA1c < 6.0%, 7.0%–7.9%, 8.0%–8.9%, 9.0%–9.9%, and≥10.0%, respectively, compared with that in HbA1c 6.0%–6.9%. Fig 1 presents the cumulative incidence curves of ESRD based on HbA1c levels. Patients with baseline HbA1c≥10.0% were at an increased risk of ESRD (log-rank test *P* < .0001).

**Table 2:** ESRD incidence and risk at different HbA1c levels.

HbA1c (%)	No of ESRD cases	Pearson-years	IDR (95% CI)^a^	Age- and gender-adjusted model HR (95% CI)	Adjusted model 1 HR (95% CI)	Adjusted model 2 HR (95% CI)
<6.0	155	35734.32	4.34 (3.65, 5.02)	1.42 (1.17, 1.73)***	2.04 (1.66, 2.51) ***	1.99 (1.62, 2.44) ***
**6.0–6.9**	**364**	**84917.23**	**4.29 (3.85, 4.73)**	**1.00**	**1.00**	**1.00**
7.0–7.9	465	90800.38	5.12 (4.66, 5.59)	1.79 (1.55, 2.07) ***	1.86 (1.59, 2.19) ***	1.86 (1.58, 2.18) ***
8.0–8.9	422	71827.18	5.88 (5.31, 6.44)	2.15 (1.86, 2.49) ***	2.12 (1.80, 2.49) ***	2.08 (1.77, 2.45) ***
9.0–9.9	365	52437.64	6.96 (6.25, 7.67)	2.70 (2.32, 3.14) ***	2.59 (2.19, 3.07) ***	2.50 (2.11, 2.96) ***
≥10	842	81506.59	10.33 (9.63, 11.03)	4.30 (3.79, 4.89) ***	4.51 (3.88, 5.24) ***	4.42 (3.80, 5.14) ***
*P* for trend				< .001	< .001	< .001

***: *P* < .001.

a: The unit is per 1,000 person-years.

IDR: incidence density rate (= number of incidence cases/person-years*1000). Adjusted model 1: age, sex, diabetes durations, smoking, drinking, obesity, hypertension, antihypertensive medication, hyperlipidemia, type of antidiabetes medication, baseline eGFR. Adjusted model 2: neuropathy, severe hypoglycemia, DKA, HHNK, cerebral vascular accident, coronary artery disease, congestive heart failure, and cancer in addition to variables in adjusted model 1.

**Fig 2:**
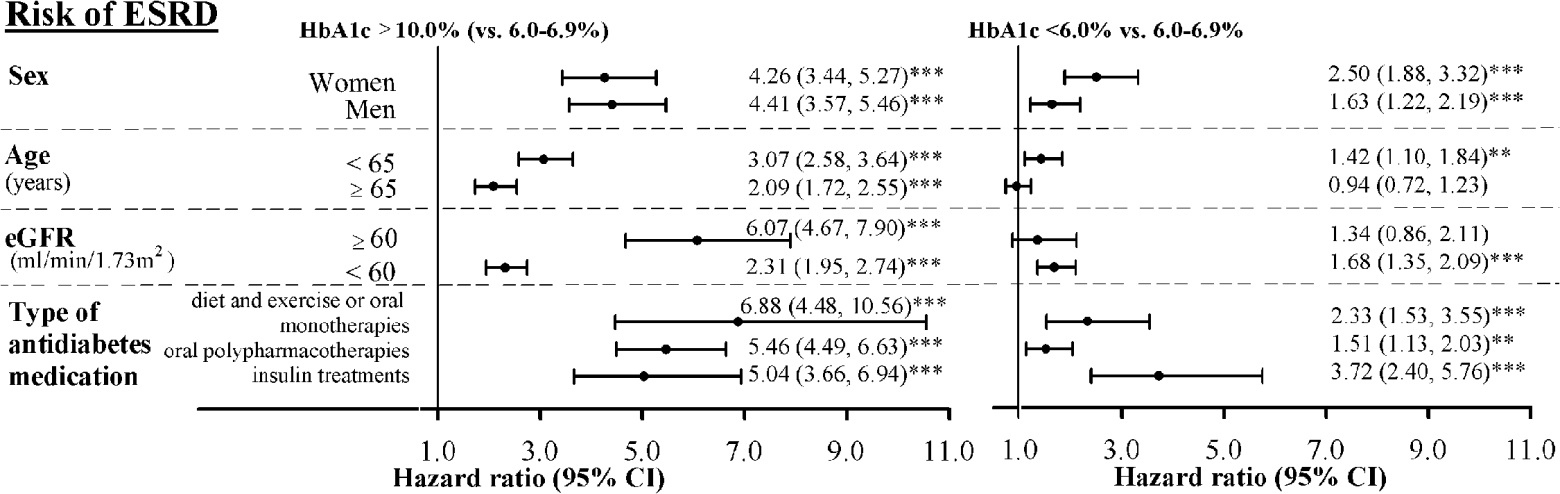
Adjusted HRs and their 95% CIs for ESRD based on HbA1c levels of≥10.0% and < 6.0%. Adjusted HRs of ESRD risk by baseline HbA1c according to sex (women or men), age (< 65 or ≥65 years), eGFR (≥60 or < 60 ml/min/1.73 m^2^), and type of antidiabetes medication (diet and exercise or oral monotherapies, oral polypharmacotherapies, and insulin treatments). The HRs were adjusted for age, sex, diabetes durations, smoking, drinking, obesity, hypertension, antihypertensive medication, hyperlipidemia, type of antidiabetes medication, baseline eGFR, neuropathy, severe hypoglycemia, DKA, HHNK, cerebral vascular accident, coronary artery disease, congestive heart failure, and cancer, except for stratified variable. Significant interaction between HbA1c and sex ($\chi_{5}^{2}$ = 105.41, *P* < .001); between HbA1c and age ($\chi_{5}^{2}$ = 38.80, *P* < .001); between HbA1c and eGFR ($\chi_{5}^{2}$ = 52.42, *P* < .001); and between HbA1c and types of antidiabetes medication ($\chi_{10}^{2}$ = 689.99, *P* < .001) on ESRD risk. **: *P* < .01; ***: *P* < .001.

Fig 2 shows the adjusted HRs and their 95% CIs for ESRD based on HbA1c levels of≥10.0% and < 6.0% stratified by sex (women or men), age ( < 65 or ≥65 y), eGFR ( ≥ 60 or < 60 mL/min/1.73 m^2^), and types of antidiabetes medication (diet and exercise or oral monotherapies, oral polypharmacotherapies, and insulin treatments). We still observed a J-shaped relationship between HbA1c and ESRD risk across these different stratification subgroups. Comparing HbA1c level ≥10.0% with 6.0%–6.9%, the ESRD risk consistently increased across various strata (all *P *< .001). There were similar trends in comparing HbA1c level < 6.0% with 6.0%–6.9%, except age≥65 years and eGFR≥60 ml/min/1.73m² subgroups. Significant interaction between HbA1c and sex ($\chi_{5}^{2}$ = 105.41, *P* < .001); interaction between HbA1c and age ($\chi_{5}^{2}$ = 38.80, *P* < .001); interaction between HbA1c and eGFR ($\chi_{5}^{2}$ = 52.42, *P* < .001); and interaction between HbA1c and types of antidiabetes medication ($\chi_{10}^{2}$ = 689.99, *P* < .001) on ESRD risk were observed.

## Discussion

We used NDCMP data and nationwide NHI claims data to estimate the incidence of ESRD from 2002 to 2011, and evaluate the association between baseline HbA1c and incident ESRD in patients with type 2 diabetes. A total of 2613 patients were diagnosed with new-onset ESRD during follow up. The lowest incidence of ESRD was 4.29 per 1000 person-years in the groups with HbA1c levels of 6.0%–6.9%; the highest ESRD incidence was 10.33 per 1000 person-years in the HbA1c of ≥ 10.0% group. A J-shaped relationship between HbA1c and ESRD risk was observed in the entire sample. Moreover, interaction between HbA1c and types of antidiabetes medication on ESRD risk was significant (*P* < .05).

A study in South Korea was performed to assess the appropriate HbA1c level for minimizing ESRD incidence in diabetic patients by using the Seoul National University Bundang Hospital database [13]. The authors demonstrated that the cumulative incidence of ESRD was directly proportional to baseline HbA1c level: 1.3% in HbA1c < 6.5%, 1.8% in HbA1c 6.5%–7.49%, and 2.7% in HbA1c≥7.5% (*P*  =  .017). The risks of ESRD in HbA1c 6.5%–7.49% and≥ 7.5% were significantly increased compared with that in HbA1c < 6.5%, respectively. Because of the limited sample size and hospital-based study design, the authors did not evaluate the effect of a more extreme cutoff point of HbA1c on ESRD risk. Our study revealed that HbA1c < 6.0% or≥7.0% increased the risk of incident ESRD in the entire sample of the Han Chinese population, with a greater variation of this effect in subgroups based on eGFR level and types of antidiabetic medication.

Our study observed a J-shaped relationship between HbA1c level and ESRD, in contrast, a population-based cohort study in Canada conducted by Shurraw et al did not observe an overall relationship between HbA1c level and ESRD [12]. They found significant interaction between HbA1c and eGFR on ESRD risk, i.e., HbA1c level > 9% was associated with increased risk of ESRD associated at higher level of baseline eGFR. In our study, we also observed the significant interaction between HbA1c and eGFR levels, which was consistent with those reported by Shurraw et al. The Canadian population-based cohort study had 2 crucial limitations: they could not control for certain potential confounders, such as the use of insulin or other medications and laboratory markers, and they could not distinguish between type 1 and type 2 diabetes [12]. Furthermore, they did not consider competing risk analysis. The traditional Cox regression does not taking competing risks into account. As for the relationship between HbA1c level and all-cause mortality, a significant linear relationship was observed in our study; the risk of mortality increased as the level of HbA1c increased (*P*  for trend: < .001, HR: 1.04, 95% CI: 1.03–1.06 for every 1% increment in HbA1c).

In previous RCTs exploring the effect of intensive glucose control intervention on DN, 2 RCTs have demonstrated that intervention targeting intensive glucose control can reduce the risk of new-onset micro- and macroalbuminuria in type 2 diabetes patients [8, 9], but 2 other RCTs have lacked evidence [10, 11]. Inconsistencies also exist regarding the relationships between strict glycemic control and ESRD [8–11]. Furthermore, Coca et al performed meta-analysis to assess the role of tight glucose control intervention in the development of surrogate and clinical renal endpoints in type 2 diabetes patients [18]. They concluded that strict glycemic control intervention could reduce the risk of micro- and macroalbuminuria, but lacked evidence related to ESRD. These RCT studies provided experimental evidence, but did not reveal the natural relationship between HbA1c and ESRD. The results of these RCTs indicate that the median HbA1c level was approximately 6.5%–7.0% in the intensive-therapy group, with 5–10 years of follow up [8–11, 19, 20], which was lower than those in the standard therapy group (approximately 7.0%–8.4%).

DN is a complex disease, and multiple mechanisms contribute to DN development. Hyperglycemia plays a pivotal role in DN development, which affects mesangial cells and glomerular injury. Mesangial cells are of critical importance to kidney function in maintaining glomerular capillary structure and regulating glomerular filtration [21]. Previous studies have demonstrated that hyperglycemia is linked to increased mesangial cell matrix production [22, 23] and mesangial cell apoptosis [24, 25]. Three mechanisms have been proposed to explain how hyperglycemia can lead to tissue damage, including nonenzymatic glycosylation, activation of protein kinase C, and acceleration of the polyol pathway [26, 27]. A possible explanation for the increased risk of ESRD for HbA1c < 6.0% is that diabetic patients with low levels of HbA1c are likely to have hypoglycemia, which results in increased risk of hyperglycemia [28]. A previous study revealed that hyperglycemia after hypoglycemia could worsen endothelial function and increase oxidative stress and inflammation [28].

The current study has several strengths. First, we used nationwide data with a large sample size, and all patients diagnosed with diabetes were invited to participate in the NDCMP, which provides a set of integrated examinations and performance measures for participants. Second, the NHI program in Taiwan provides continuing universal coverage for the entire population, which avoids selection bias. Third, using NHI datasets eliminated the need to minimize the number of cohort participants lost to follow up. In addition, we easily obtained a large sample of geographically dispersed patients. Fourth, we defined the new-onset ESRD cases based on the Registry for Catastrophic Illness Patients Database, which resulted in the high ascertainment validity of the ESRD cases. This could minimize ESRD misclassification bias. Finally, we controlled for numerous crucial clinical and demographic factors, such as laboratory markers, types of antidiabetes medication, antihypertensive medication, and diabetes-related comorbidities, which could minimize the effect of potential confounders.

Nevertheless, three limitations deserve to be mentioned. First, we did not conduct repeated measurements of HbA1c for all the study participants within 1 year of the follow-up period to reduce the random error of once HbA1c measurement. Using approximately 60% of the participants, the mean value of HbA1c measurements obtained within 1 year is a significant predictor of ESRD risk, demonstrating a J-shaped relationship between HbA1c and ESRD risk. Second, we also did not acquire the HbA1c measurement for the later follow-up period; thus, we could not examine the effect of time-varying HbA1c on ESRD risk. Finally, some patients (n = 250, 0.48%) with CKD stage 5 in baseline assessments were included. Prior studies have provided evidence that HbA1c levels were false low [29–31] due to renal anemia and reduced lifespan of erythrocytes in patients with chronic dialysis. To rule out the impact of chronic dialysis on our findings, we have excluded patients with chronic dialysis at baseline. Thus, the possibility that our findings about the association between low HbA1c level and higher incidence of ESRD was explained by chronic dialysis can be minimized.

## Conclusion

In conclusion, our data suggest that a low or high HbA1c value is linked to increased ESRD risk in the population of Chinese patients with type 2 diabetes in Taiwan. Our findings are consistent with the literature indicating that HbA1c level plays a crucial role in ESRD development. Diabetes care has focused on preventing hyperglycemia, but has ignored hypoglycemia. Because a low level HbA1c has the potential risk of causing ESRD to develop, appropriate glycemic control is essential for minimizing ESRD risk, which should be emphasized in diabetes care. Clinicians need to pay attention to HbA1c results on DN.

## Data Availability Statement

All relevant data are within the paper. Data are from the Taiwan Diabetes Study whose authors may be contacted at tcli@mail.cmu.edu.tw.

## References

[1] Collins AJ, Foley RN, Chavers B, Gilbertson D, Herzog C, Johansen K, et al United States Renal Data System 2011 Annual Data Report: Atlas of chronic kidney disease & end-stage renal disease in the United States. Am J Kidney Dis. 2012;59(1 Suppl 1):A7, e1–420. Epub 2011/12/30. 10.1053/j.ajkd.2011.11.015 S0272-6386(11)01571-X [pii]. . 22177944

[2] The global dominance of diabetes. Lancet. 2013;382(9906):1680 10.1016/S0140-6736(13)62390-9 . 24267986

[3] Yang WC, Hwang SJ. Incidence, prevalence and mortality trends of dialysis end-stage renal disease in Taiwan from 1990 to 2001: the impact of national health insurance. Nephrol Dial Transplant. 2008;23(12):3977–82. Epub 2008/07/17. 10.1093/ndt/gfn406 gfn406 [pii]. . 18628366

[4] The Report of National Health Insurance, 2007 [in Chinese]. Bureau of National Health Insurance (BNHI), Department of Health, Republic of China (Taiwan). 2007. Available from: http://www.nhi.gov.tw/Resource/webdata/Attach_8965_2_%E9%87%8D%E5%A4%A7%E5%82%B7%E7%97%85%E9%A0%98%E8%AD%89%E6%95%B8%E8%A1%A818-%E9%99%84%E4%BB%B68.pdf.

[5] The Report of National Health Insurance, 2011 [in Chinese]. Bureau of National Health Insurance (BNHI), Department of Health, Republic of China (Taiwan). 2011. Available from: http://www.nhi.gov.tw/webdata/webdata.aspx?menu=17&menu_id=661&WD_ID=685&webdata_id=3627.

[6] Standards of medical care in diabetes—2012. Diabetes Care. 2012;35 Suppl 1:S11–63. Epub 2012/01/04. 10.2337/dc12-s011 35/Supplement_1/S11 [pii]. . 22187469 PMC3632172

[7] Tu ST, Chang SJ, Chen JF, Tien KJ, Hsiao JY, Chen HC, et al Prevention of diabetic nephropathy by tight target control in an asian population with type 2 diabetes mellitus: a 4-year prospective analysis. Arch Intern Med. 2010;170(2):155–61. Epub 2010/01/27. 10.1001/archinternmed.2009.471 170/2/155 [pii]. . 20101010

[8] Ismail-Beigi F, Craven T, Banerji MA, Basile J, Calles J, Cohen RM, et al Effect of intensive treatment of hyperglycaemia on microvascular outcomes in type 2 diabetes: an analysis of the ACCORD randomised trial. Lancet. 2010;376(9739):419–30. 10.1016/S0140-6736(10)60576-4 . 20594588 PMC4123233

[9] Perkovic V, Heerspink HL, Chalmers J, Woodward M, Jun M, Li Q, et al Intensive glucose control improves kidney outcomes in patients with type 2 diabetes. Kidney international. 2013;83(3):517–23. 10.1038/ki.2012.401 . 23302714

[10] Duckworth W, Abraira C, Moritz T, Reda D, Emanuele N, Reaven PD, et al Glucose control and vascular complications in veterans with type 2 diabetes. The New England journal of medicine. 2009;360(2):129–39. 10.1056/NEJMoa0808431 . 19092145

[11] Intensive blood-glucose control with sulphonylureas or insulin compared with conventional treatment and risk of complications in patients with type 2 diabetes (UKPDS 33). UK Prospective Diabetes Study (UKPDS) Group. Lancet. 1998;352(9131):837–53. Epub 1998/09/22. S0140673698070196 [pii]. . 9742976

[12] Shurraw S, Hemmelgarn B, Lin M, Majumdar SR, Klarenbach S, Manns B, et al Association between glycemic control and adverse outcomes in people with diabetes mellitus and chronic kidney disease: a population-based cohort study. Arch Intern Med. 2011;171(21):1920–7. 10.1001/archinternmed.2011.537 . 22123800

[13] Oh SW, Kim YC, Koo HS, Jin DC, Na KY, Chae DW, et al Glycated haemoglobin and the incidence of end-stage renal disease in diabetics. Nephrol Dial Transplant. 2011;26(7):2238–44. 10.1093/ndt/gfq707 . 21098657

[14] Levey AS, Stevens LA, Schmid CH, Zhang YL, Castro AF 3rd, Feldman HI, et al A new equation to estimate glomerular filtration rate. Annals of internal medicine. 2009;150(9):604–12. 19414839 10.7326/0003-4819-150-9-200905050-00006 PMC2763564

[15] Introduction to the National Health Insurance Research Database, Taiwan National Health Research Institutes (NHRI) Available from: http://w3.nhri.org.tw/nhird//date_01.html.

[16] Ministry of Health and Welfare. Available from: http://law.moj.gov.tw/LawClass/LawAll.aspx?PCode=L0060006.

[17] Lunn M, McNeil D. Applying Cox regression to competing risks. Biometrics. 1995;51(2):524–32. . 7662841

[18] Coca SG, Ismail-Beigi F, Haq N, Krumholz HM, Parikh CR. Role of intensive glucose control in development of renal end points in type 2 diabetes mellitus: systematic review and meta-analysis intensive glucose control in type 2 diabetes. Arch Intern Med. 2012;172(10):761–9. 10.1001/archinternmed.2011.2230 22636820 PMC3688081

[19] Action to Control Cardiovascular Risk in Diabetes Study Group, Gerstein HC, Miller ME, Byington RP, Goff DC Jr, Bigger JT, et al Effects of intensive glucose lowering in type 2 diabetes. The New England journal of medicine. 2008;358(24):2545–59. 10.1056/NEJMoa0802743 . 18539917 PMC4551392

[20] ADVANCE Collaborative Group, Patel A, MacMahon S, Chalmers J, Neal B, Billot L, et al Intensive blood glucose control and vascular outcomes in patients with type 2 diabetes. The New England journal of medicine. 2008;358(24):2560–72. 10.1056/NEJMoa0802987 . 18539916

[21] Dronavalli S, Duka I, Bakris GL. The pathogenesis of diabetic nephropathy. Nature clinical practice Endocrinology & metabolism. 2008;4(8):444–52. 10.1038/ncpendmet0894 . 18607402

[22] Harris RD, Steffes MW, Bilous RW, Sutherland DE, Mauer SM. Global glomerular sclerosis and glomerular arteriolar hyalinosis in insulin dependent diabetes. Kidney international. 1991;40(1):107–14. . 1921145 10.1038/ki.1991.187

[23] Heilig CW, Concepcion LA, Riser BL, Freytag SO, Zhu M, Cortes P. Overexpression of glucose transporters in rat mesangial cells cultured in a normal glucose milieu mimics the diabetic phenotype. The Journal of clinical investigation. 1995;96(4):1802–14. 10.1172/JCI118226 7560072 PMC185817

[24] Mishra R, Emancipator SN, Kern T, Simonson MS. High glucose evokes an intrinsic proapoptotic signaling pathway in mesangial cells. Kidney international. 2005;67(1):82–93. 10.1111/j.1523-1755.2005.00058.x . 15610231

[25] Lin CL, Wang JY, Huang YT, Kuo YH, Surendran K, Wang FS. Wnt/beta-catenin signaling modulates survival of high glucose-stressed mesangial cells. Journal of the American Society of Nephrology: JASN. 2006;17(10):2812–20. 10.1681/ASN.2005121355 . 16943306

[26] Friedman EA. Advanced glycation end-products in diabetic nephropathy. Nephrol Dial Transplant. 1999;14 Suppl 3:1–9. . 10382974 10.1093/ndt/14.suppl_3.1

[27] Porte D Jr, Schwartz MW. Diabetes complications: why is glucose potentially toxic? Science. 1996;272(5262):699–700. . 8614830 10.1126/science.272.5262.699

[28] Ceriello A, Novials A, Ortega E, La Sala L, Pujadas G, Testa R, et al Evidence that hyperglycemia after recovery from hypoglycemia worsens endothelial function and increases oxidative stress and inflammation in healthy control subjects and subjects with type 1 diabetes. Diabetes. 2012;61(11):2993–7. 10.2337/db12-0224 22891214 PMC3478543

[29] Inaba M, Okuno S, Kumeda Y, Yamada S, Imanishi Y, Tabata T, et al Glycated albumin is a better glycemic indicator than glycated hemoglobin values in hemodialysis patients with diabetes: effect of anemia and erythropoietin injection. Journal of the American Society of Nephrology: JASN. 2007;18(3):896–903. 10.1681/ASN.2006070772 . 17267743

[30] Cohen RM, Smith EP. Frequency of HbA1c discordance in estimating blood glucose control. Current opinion in clinical nutrition and metabolic care. 2008;11(4):512–7. 10.1097/MCO.0b013e32830467bd . 18542015

[31] Riveline JP, Teynie J, Belmouaz S, Franc S, Dardari D, Bauwens M, et al Glycaemic control in type 2 diabetic patients on chronic haemodialysis: use of a continuous glucose monitoring system. Nephrol Dial Transplant. 2009;24(9):2866–71. 10.1093/ndt/gfp181 . 19389864

